# Tumor clonal status predicts clinical outcomes of lung adenocarcinoma with EGFR-TKI sensitizing mutation

**DOI:** 10.7150/jca.32897

**Published:** 2019-08-29

**Authors:** Eun Young Kim, Sang Hoon Lee, Arum Kim, Taehee Kim, Yoon Soo Chang

**Affiliations:** Department of Internal Medicine, Yonsei University College of Medicine, Seoul, Republic of Korea

**Keywords:** clonality, EGFR-TKI sensitizing mutation, lung adenocarcinoma, heterogeneity, NGS

## Abstract

**Objectives**: Intratumoral heterogeneity is one of major causes of resistance to therapeutic. Here, we evaluated clonal status, which may reflect intratumoral heterogeneity, in lung adenocarcinoma with EGFR-TKI sensitizing mutation (mEGFR) and its clinical implications.

**Materials and Methods**: Customized panel comprised of 71 solid tumor-associated genes were applied to the 77 surgically resected lung adenocarcinoma having mEGFR with curative aim. For comparison, whole exome sequencing (WES) data of 45 TCGA-LUAD with mEGFR were extracted from the GDC dataportal. Clonal status was estimated from the allele frequency of the mutated genes using the Maftools package.

**Results**: In the study cohort, the number of mutations per case detected by customized panel was 5 [4-8], and the number of total mutations or subtypes of mutations was not related clinical parameters, including size of tumor and pStage. The number of subclones showed positive correlation with the maximum diameter of primary tumor (Spearman's rho=0.273, P-value = 0.026). Disease-free survival (DFS) was significantly shorter among cases wherein tumors comprised two or more subclones than the cases in which tumors were comprised of one clone (P-value = 0.006, Log-rank test), and multivariate analysis indicated that the number of subclones was an independent determinant of DFS. In the WES of TCGA-LUAD mEGFR, the characteristics of the mutations did not show significant relationship with clinical parameters whereas the cases with clones with two or more showed poorer overall survival than those with one clone (P-value = 0.038, Log-rank test).

**Conclusions**: Number of subclones comprising the primary lesion was positively correlated with tumor size, and was an independent factor affecting clinical outcome, showing that a description of tumor clonality may be helpful for understanding of disease status.

## Introduction

Globally, 2.1 million lung cancer cases were newly diagnosed in the year 2018, and 1.8 million patients died of this devasting disease during the same period 1. These estimates are similar to those of Republic of Korea, showing that 25,780 new lung cancer cases were diagnosed during the year 2016 (men, 69.0%; women, 31.0%), ranked at fourth common cancer by incidence. In addition, it has been the leading cause of cancer death since 1999 2. These findings demand that a more precise understanding of the biology of lung cancer and meticulous management are required for those who are at risk of recurrence or treatment failure.

EGFR activating mutation is one of the most common drivers in the non-small cell lung cancer (NSCLC) in the Far East Asia. Completely resected stage II~IIIA lung adenocarcinoma with EGFR-tyrosine kinase (TKI) sensitizing mutation (mEGFR) is treated with cisplatin-based adjuvant chemotherapy, which is similar to other non-small cell lung cancers irrespective to the EGFR mutation status. This adjuvant therapy shows significantly improved disease-free survival (DFS) and overall survival (OS) compared with those being observed after complete resection 3. However, after the initial planned treatment, a considerable number of patients experience disease recurrence and are treated with EGFR-TKI and ultimately, they experience treatment failures due to various mechanisms 4. The 5-year survival rate in this group is only 15-30% 2. The current lung cancer staging system is an important metric for predicting the clinical outcomes from NSCLC and selecting treatment modalities. Because the indicators used to determine tumor stage are composed of only anatomical parameters, patients with the same stage often have different clinical outcomes. For example, among the lung adenocarcinoma with mEGFR, those with L858R mutation and those with EGFR exon 19 deletion encompassing LREA motif show different mutation characteristics and signature patterns 5. These findings suggest that the presence of unmet medical needs that could predict disease relapse reflecting intratumoral heterogeneity. As if the treatment paradigm of advanced NSCLC has changed based on the combination of histology, genomic alteration and PD-L1 expression, the strategy modification is also required in the precision therapy based on the intratumoral heterogeneity.

A clonal succession model in which a dominant driver mutation generates a homogenous tumor by purifying competing clones through a selective sweep is challenged by the high extent of clonal diversity observed in various carcinomas 6-8. Taken together with the fact that topological separation limits dominant clones' ability to suppress less fit clones, tumor microenvironmental heterogeneity and changes in selection pressure can enhance clonal diversity. Heterogeneity among cancer cells within a tumor is thought to be one of the major sources of response variation, including resistance to the therapeutic agents and treatment failure. Cancer phenotypic heterogeneity is due to substantial genetic changes in cancer cells, epigenetic aberrations, and interaction with abnormal and heterogonous microenvironments 9. Although genetic heterogeneity may not be a primary determinant of intratumoral phenotypic heterogeneity, it plays a key role in somatic evolution during tumor progression and drug resistance because of its heritability.

It may be important to estimate the degree of intratumoral heterogeneity before initiating treatment. Methods for estimating intratumoral heterogeneity include: (1) identification of intratumor distribution of pre-identified markers 10-12; (2) elaborate multi-regional tumor dissection 10, 11, 13, 14; (3) ultradeep sequencing 15; (4) mutant-allele tumor heterogeneity (MATH) score, which is calculated from the ratio of the width to the center of the distribution of the mutant-allele fraction of the tumor-specific mutated loci 16; and (5) inferring the number of subclones using next-generation sequencing (NGS). Most of these methods are not only technically difficult but are also practically difficult to apply in medical settings. Among them, MATH and inferring subclone number may be the most feasible to apply clinically.

In this study, seventy-seven Korean lung adenocarcinomas with mEGFR were selected and the clonal status, which may reflect intratumoral heterogeneity, was estimated from the allele frequency of the mutated tumor specific genes using a customized panel. For comparison, whole exome sequencing (WES) and masked copy-number variation (CNV) data of 45 TCGA-LUAD with mEGFR were extracted from the GDC dataportal. We also discussed the clinical significance of this approach in this homogenous subset of lung adenocarcinoma with mEGFR.

## Materials and Methods

**Study subjects.** A total of 77 lung adenocarcinoma tissues that met the following criteria were randomly selected from the tissue archives of two affiliated hospitals, Severance Hospital and Gangnam Severance Hospital, of Yonsei Medical Center (study cohort): (1) pathologically confirmed lung adenocarcinoma, (2) underwent curative aim surgical resection, (3) confirmation of informed consent for sequencing of major cancer related genes, (4) presence of the mEGFR in the tumor identified by sanger sequencing or PNA clamping method, and (5) confirmation of informed consent for tissue collection. The EGFR-TKI sensitizing mutations are defined as a point mutation in the EGFR exon 21, which substitutes an arginine for a leucine (L858R), in-frame deletions (encompassing 4 amino acid residues 747 to 750 LREA motif) of the EGFR exon 19, G719X point mutation(encoded by exon 18), and L861Q point mutation (exon 21)^17^. This study was approved by the IRB of Gangnam Severance Hospital (IRB #3-2017-0059) and it complied with the Declaration of Helsinki guidelines (http://www.wma.net/en/30publications/10policies/b3/index.html) and the Korean GCP guidelines. For comparison, publicly available data were extracted from the GDC Data Portal of The Cancer Genome Atlas (TCGA; https://cancergenome.nih.gov/). Of the 585 TCGA-LUAD cases, 502 had both SNV information from VarScan2 and clinical information, making them eligible for our analyses and 48 cases harbor mEGFR. Among them, 3 cases, TCGA-17-Z047, TCGA-17-Z032, which do not have clinical information, and TCGA-55-8506, which has exceptionally high number of mutation (total number of mutation = 1970 among 5606 was excluded **(Supplementary table 1).** TCGA-55-8506 alone has 1970 mutations that accounted for 35.1% of the 5606 mutations of TCGA-LUAD with mEGFR in total 48 cases.

**NGS.** A 0.62 Mb customized NGS panel containing 71 major cancer genes was constructed and sequenced using the Ion S5 NGS system (Thermo Fisher Scientific) (**Supplementary table 2**). To extract cancer-enriched gDNA, paraffin-embedded tissue samples were loaded onto silanated slides at 4-μm-thick sections. Each slide was lightly stained with H&E, and cancer cell enriched areas were selected after comparing them with permanently stained slides marked by an independent lung pathologist; then the slides were scrapped with clean blades. gDNA was extracted using a QIAamp DNA FFPE Tissue Kit (Qiagen, Valencia, CA, USA), and 50 ng of extracted DNA was reacted with fragmentizer for 12 to 50 minutes (Archer). The section of cut DNA was blunted and 5' phosphorylated with an end-repair enzyme (Archer). The end of the DNA strand was barcode-ligated via a reaction with an MBC adapter (Archer) for 15 minutes, and then the first and second PCRs were performed using a primer set for the selected target genes. After measuring the prepared library with Qubit^Ⓡ^, 50 pmol of sample was obtained and mixed with mineral oil to normalize with beads. The sample was applied to a 540 Chip (Life Technology) and sequenced with the S5 sequencer (Life technology). The results were then analyzed with Archer Analysis 5.1 software (Archer) and the median sequencing depth was 308X [164.5 ~ 738.5].

**Calculations of subclone numbers and MATH scores.** To estimate the number of subclones and MATH scores, the InferHeterogeneity function of the Maftools package was used, which estimates the number of subclones by clustering of the variant with the similar allele frequencies 18. The MATH score was obtained using Mroz and Rocco's method 16. MATH score for each tumor were calculated from the median values of mutant-allele fractions in the median absolute deviation (MAD) and tumor-specific mutated loci.

**Statistical analyses.** Distribution of variables was examined using the Shapiro-Wilk test. We assessed differences in the distribution of continuous variables between two independent samples using the Mann-Whitney U test and we used the Kruskal-Wallis test to compare the medians of three or more groups. Analyses were performed using SPSS version 23 (SPSS Inc., Chicago, IL, USA). All statistical tests were two-sided, and a P-value <0.05 was considered statistically significant.

## Results

**Number of variants in the study cohort.** We performed targeted sequencing of 71 major solid cancer-related genes on a set of 77 lung adenocarcinoma samples. The demographic characteristics of the study cases are described in Table 1. The median patient age was 62.0 years [54.0 ~ 69.0 years], and the male-to-female ratio was 1: 2.85. The study set was comprised of 36 cases of stage I, 20 stage II, 21 stage III and none of the cases had taken EGFR-TKI before documentation of recurrence.

**Figure 1:**
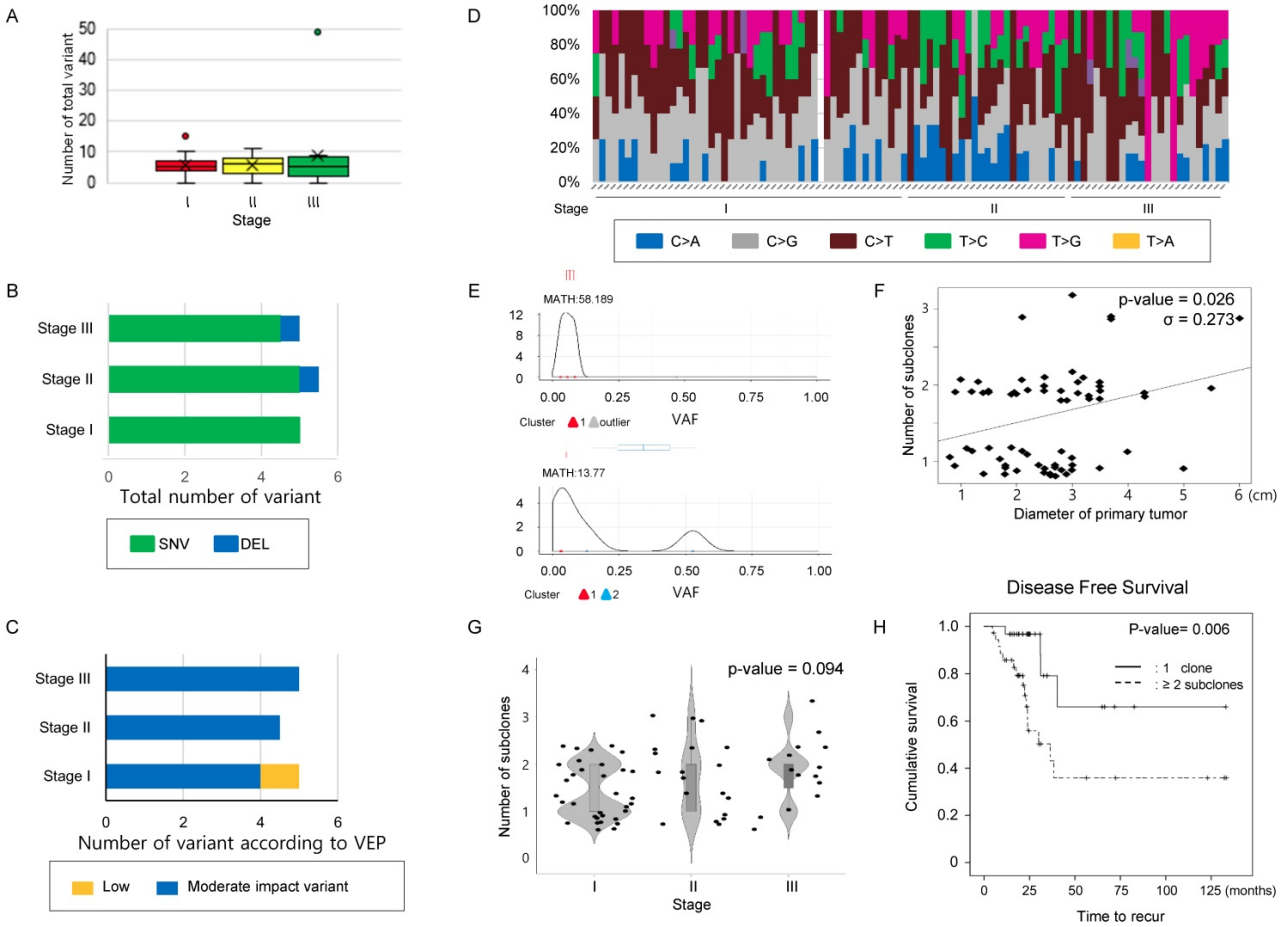
** Comparison of mutation characteristics and number of clones according to the stage in the study cohort.** (A) The number of total mutations, (B) the number of mutations categorized into SNV, INS, and DEL, and (C) those classified according to VEP—high, moderate, low impact variant, and modifier were compared according to stage. (D) A histogram showing substitution type of SNVs per individual case. (A~D) The number of total mutations and that of subcategorized mutations were not statistically significant according to stage. The difference was estimated with the Kruskal-Wallis rank-sum test. (E) Representative figure used for clonality estimation. These figures were derived from “inferHeterogeneity” and “plotClusters” function in the Maftools package. (F) Scatter plot showing the relationship between the maximum diameter of the primary tumor and number of subclone. The maximum primary tumor diameter and the number of subclones were positively correlated (Pearson's correlation efficiency, σ = 0.273, P-value = 0.026). (G) A violin plot showing the number of subclones according to the stages. P-value was obtained by chi-square test. (H) The cases were classified into two groups, where the primary tumors consisted of either one clone and two or more subclones, and DFS were calculated using the Kaplan-Meier estimator. The cases with 2 or more subclones showed shorter DFS than those with one clone. P-values were obtained by the log-rank test.

A mean 5.0 [4.0 ~ 7.0] mutations was detected per case in our 71-gene panel. The total number of mutations (Figure 1A) and the number of mutations classified as SNV, INS, or DEL (Figure 1B) were not significantly different according to stage (Table 2). Next, the variants were classified into modifier, high, moderate, or low impact variants using the variant effect predictor (VEP), as presented in the TCGA (Supplementary table 3) and were analyzed according to stages. Low impact variants were frequently observed in stage 1, but the remaining mutations did not distribute differently across the stages (Table 2 and Figure 1C). Finally, transversion and transition mutations and the ratio of the two were compared according to the stage, and no significant differences were observed in the mutation numbers and the ratio according to stage (Table 2 and Figure 1D). These findings suggest that neither the number of simple mutations nor the predictions from biological effects of the variants are related to tumor stage; thus, they may have limitations for predicting intratumoral heterogeneity.

**Increase in subclone number according to the size of tumor in the study cohort.** Next, to infer that the number of subclones could reflect disease status, we estimated that the number of subclones in the single primary lesions from recruited cases (Figure 1E). When the number of subclones was compared to primary lesion size, there was a significant positive correlation between maximum primary lesion diameter and number of subclones constituting the lesion (σ = 0.273, P-value = 0.026, Figure 1F). When comparing the number of subclones according to stage, there was a trend increasing in the number of subclones with increasing stage, but it did not reach statistical significance (P-value= 0.094, chi-square test, Figure 1G and Supplementary table 4). Additionally, we examined the relationship between tumor size/stage and MATH score, which was calculated from the median absolute deviation (MAD) and the median of the mutant allele fraction value of tumor-specific mutated loci 16. The mean MATH score of the cases was 55.282 [29.911 ~ 84.503] (Supplementary Figure 1A and Supplementary table 4). Comparison of MATH score among the different stage group revealed that the MATH score of stage III group was higher than that of stage II. On one hand, the MATH score of stage I even tended to be higher than that of stage II, and there was no significant association between MATH score and other parameters such as tumor size and number of subclones, suggesting that MATH score may have limitation to pursue clinical implications in this subset of lung cancer.

**Number of subclones in the primary lesion determines DFS.** To evaluate the effect of the number of subclones estimated from the NGS results on clinical outcomes, we performed Kaplan-Meier tests to estimate the effect of parameters on DFS, including the number of subclones. The median follow-up duration was 72.5 months (95% CI; 49.74 ~ 95.26 months), and 29 (29.9%) cases experienced lung cancer recurrence during the follow-up period. A total of 23 (23.7%) patients died during the follow-up period. Univariate analyses were used to identify main effects from the following variables: age, sex, smoking history, number of subclones constituting the primary lesion, and stage; only stage and number of subclones had a significant effect on DFS (Figure 1H, and Table 3). There was no significant relationship with regard to multicollinearity verification between the number of subclones and other factors that could affect recurrence. Only the number of subclones and stage were major determinants of DFS in multivariate analysis. The effect of number of subclones on the OS was also evaluated, which did not affect the OS (Supplementary Figure 1B).

**Figure 2:**
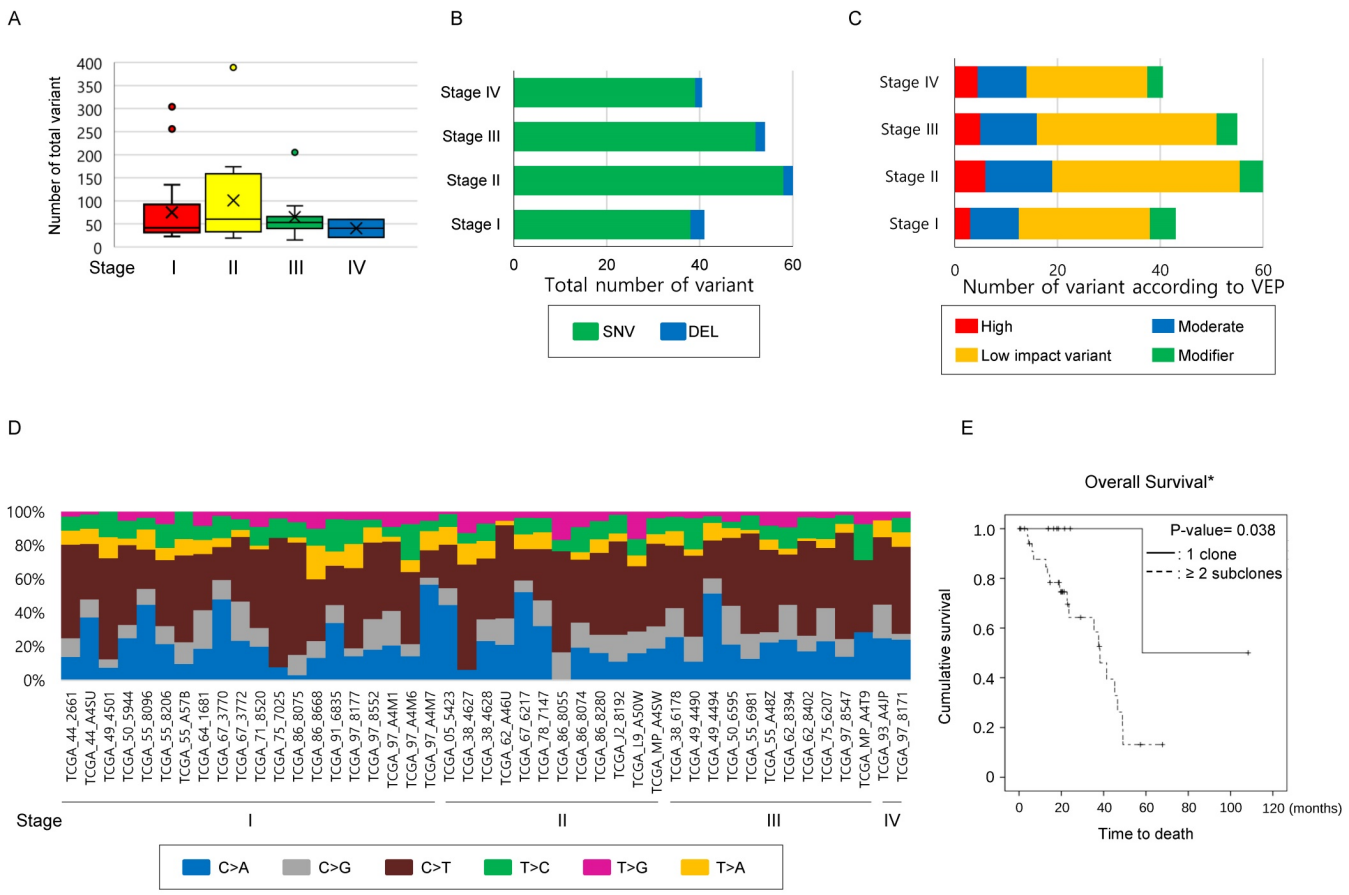
** Comparison of mutational characteristics according to the stage in the TCGA-LUAD with mEGFR cohort.** The 45 TCGA-LUAD cases, which had WES mutation information by VarScan2, mEGFR, and clinical information including stage were recruited from the GDC Data Portal (https://portal.gdc.cancer.gov/) and (A) The number of total mutations, and (B) the number of mutations classified into SNV, INS, and DEL were compared according to stage. (C) All mutations detected were classified according to VEP—high, moderate, low impact variant, and modifier—and compared according to the stage. (D) A histogram showing substitution type of SNVs per individual case. (A~D) The number of total mutations and that of subcategorized mutations were not statistically significant according to the stage. The differences were estimated by the Kruskal-Wallis rank-sum test. (E) Survival analysis according to the number of subclones in TCGA-LUAD mEGFR cohort. The cases with mEGFR were classified into two groups, having one clone and two or more subclones, and then OS was compared between the groups using the Kaplan-Myer estimator. Cases with two or more subclones detected in the primary lesion showed significantly shorter OS than those whose tumors harbored one clone. *Note that in the GDC Data Portal only OS was available. P-values were obtained using the log-rank test.

**Number of mutations has limitations in providing clinical implications in the TCGA-LUAD mEGFR cohort.** To clarify whether the findings observed in the study cohort could be identified in the TCGA-LAUD cases with mEGFR, we recruited WES data from VarScan2. The mean age of this cohort was 66.28 ± 9.53 years and comprised of 11 males and 34 females. The Stage I was 20, stage II 12, stage III 11, and stage IV 2. The median number of non-synonymous mutations was 51 [32.0 ~ 79.0] per TCGA-LUAD mEGFR case which was not significantly different according to stage (Figure 2A, Table 4), and when these were classified into SNV, INS and DEL, there was no significant difference among the stage groups (Figure 2B). When the variants were divided according to VEP, the number of high impact variants, which is expected to affect structural changes and biological functions of the protein, was 5 [3 ~ 8] per case, and that of moderate impact variants was 28.0 [19.0 ~ 46.0] per case. Stage did not influence the number variants classified by the VEP (Figure 2C). Finally, we examined the differences in SNV classes among the cases according to their stage. In this subset of lung adenocarcinoma, C>T substitution were most frequently observed, occurring in 21 [13 ~ 35.0] per case followed by C>A, C>G, T>C, T>A, and T>G substitutions. We also found no difference among SNV classes or number and ratio of transition and transversion mutations according to stage (Figure 2D). In summary these findings were similar to the findings from the study cohort, indicating the number of mutations was not highly correlated with the primary clinical parameters.

**Number of subclones in the primary lesion determines OS in the TCGA-LUAD mEGFR.** To clarify that the increased number of subclones are related poor clinical outcome, 45 TCGA-LUAD mEGFR cases, which have clinical information, WES data from VarScan2, and masked CNV files, were recruited. Among the clinical data of TGCA-LUAD, information on the treatment, DFS and, PFS was not provided and, therefore, only the OS was analyzed. When estimating the number of clones, masked CNV files was used to exclude mutations that locates on the CNV area. In the TCGA-LUAD mEGFR cases, the median number of the subclones was two [1.75 ~ 2.0]. The cases with two or more subclones had significantly poorer prognoses compared with those with one clone, indicating that the more clones present, the worse the clinical outcome (Figure 2E, P-value = 0.038, Log-rank test). Multivariate analysis revealed that among the age, gender, stage, smoking history and number of subclone, the number of subclone was independent determinant of OS with smoking status. Multicollinearity test showed that there was no interaction observed between the tested variables. Taken together, these findings suggest that the number of subclones is one of the poor prognostic factors of lung adenocarcinoma with mEGFR.

## Discussion

Relentless growth and increasing heterogeneity from onset may be the main causes of treatment failure, but indicators that properly reflect heterogeneity are not widely used in the medical field. This study might have important clinical implications because we evaluated the number of subclones as an index reflecting heterogeneity using data obtained from a targeted NGS panel in a subset of homogenous NSCLC cases.

In the study cohort and TCGA-LUAD mEGFR cohort, the total number of mutations and the number of mutations classified by nature did not show much correlation with clinical parameters. To clarify that these findings could be observed in the entire TCGA-LUAD set, we recruited 582 cases with stage I ~ IV, mutation data from VarScan2, and masked CNV files among 585 TCGA-LUAD cases. The median number of non-synonymous mutations was 197 [79 ~ 424.0] per TCGA-LUAD case, which was not significantly different according to stage (Supplementary table 5 and Supplementary figure 2A). When these were classified either into SNV, INS and DEL, or divided according to VEP there was no significant difference among the stage groups (Supplementary figure 2B~C). Finally, we examined the differences in SNV classes among the cases according to their stage. Consistent with well-known lung-cancer characteristics, C>A mutations were most frequently observed, occurring in 76 [16 ~ 180.5] of the 6 SNV classes, followed by C>T, C>G, T>A, T>C, and T>G substitutions. We also found no difference among SNV classes or number and ratio of transition and transversion according to stage (Supplementary figure 2D).

In this study, we evaluated tumor clonality using a targeted NSG panel comprised of 71 solid tumor-associated genes, which may harbor limitations, such as: (1) the number of genes were different from that of WES, and (2) CNV and (3) chromosomal ploidy were not reflected. Despite differences between the two platforms, the major gene that showed altered VAF and had a major influence on estimating the number of subclones was TP53 in both cohorts, and the contribution of the remaining genes was minimal. Specifically, the frequently commutated genes in the study cohort were TP53 followed by FBXW7 and KRAS, whereas in the TCGA-LUAD mEGFR cohort the order of frequently commutated genes was TP53 followed by CSMD3, and MUC16. These findings suggested that the WES may not absolutely required for clonality estimation in cancer and that further studies and efforts are required to construct a cost effect panel with frequently mutated, clinically significant genes in the production of the targeted sequencing panel. To elucidate the influence of mutations located in the copy number altered lesion on the estimation of subclone numbers, we compared the number of subclones either removing the mutations located at the position where the CNV was present or not considering CNV in the calculation. Both mean numbers of subclones calculated were 2 [2 ~ 3], regardless of whether we removed mutations located at the position where the CNV was present or did not consider CNV in the calculation (σ= 0.756, P-value <0.001, Pearson's correlation test). These findings suggest that there is little difference in the number of subclones between by removing the mutations located where the CNV exists and not removing. TCGA-LUAD does not provide information on disease relapse but information on survival period, which make it difficult to directly compare the results obtained in the two cohort studies. After initial diagnosis of the lung adenocarcinoma with mEGFR, surgical resection or targeted therapy is applied based on the disease stage. Because the various treatment modalities are applied when disease relapse, there are various variables that limit OS as a good surrogate maker representing the biology of the tumor. As evaluated in the study cohort, evaluating DFS after homogenous treatment such as surgical resection of curative aim would be more meaningful to find the effect of tumor clonality on the disease nature and its clinical implication.

In our analyses of the 502 TCGA-LUAD cases that had clinical information, the number of subclones did not influence statistically significant differences in OS. However, when we gradually confined the analyses to the earlier-stage TCGA-LUAD cases, the cases with three or more subclones showed a distinct tendency to have worse OS than those with one or two subclones (Supplementary Figure 3). We inferred the reason that the differences in the OS were gradually evident toward the earlier stages might be that the NGS results from earlier stages might be obtained from a majority proportion of the tumor than those from advanced stage.

MATH is an index of intratumoral heterogeneity that attracts attention due to its theoretical background and the convenience of making calculations using the NGS panel. It can be calculated based on the assumption of (1) heterozygous loci, (2) no CNA, and (3) no mixture of normal tissues 16. Mroz et al. suggested that by using the median of the mutant-allele fraction in the tumor-specific mutated loci as a denominator, the effect of normal tissue contamination can be offset by the MATH value. They also suggested that the effect of outliers derived from the mutant allele frequently observed on the CNV site, low fraction loci with incorrect values, and the high-fraction loci from most cells, including normal cells, could be ruled out 16. The relationship between MATH values and clinical outcome/stage could not be observed in the TCGA-LUAD data or in our study dataset. Furthermore, the MATH scores from this study cohort from targeted NGS showed a significant distribution difference when compared with those from the TCGA-LUAD. The relationship between MATH value and clinical outcome was not significant in either group, suggesting that the clinical applicability of this method should be further supplemented.

Nowadays, NGS is widely used in various medical fields. Besides on the report on the SNV, it is required to widen its clinical usefulness. For example, the TMB obtained from WES has been suggested as an index for predicting the therapeutic response to immunotherapy for NSCLC 19. Clonality is a simple parameter that can be easily calculated from NGS data and might have clinical significance; this approach requires further validation from a large number of cases. When evaluating intratumoral heterogeneity and clonality, it is prudent to consider whether a small biopsy is appropriate or if a new approach is needed. Additional studies are also needed to compare the differences among results obtained from high-cost measurements, including WES, CNV, and chromosome ploidy, as well as those from targeted sequencing.

## Figures and Tables

**Table 1:** Demographic characteristics of the study cohort.

Characteristics		n = 77
Age (years)		62.0 [54.0 ~ 69.0]
Sex		
	Male	20
	Female	57
Smoking status		
	Ever smoker	56
	Never smoker	21
Pack years (years)		24.54 ± 14.92
pStage	I	36
	II	20
	III	21
MATH score		62.51 [30.28 ~ 87.48]
Number of subclones	1	31
	2	31
	3	5

**Table 2:** Comparison of variants according to stage in the study cohort.

			Stage I	Stage II	Stage III	P-value*
Study cohort	Total variant		5.0 [4.0-6.0]	6.0 [3.0-8.0]	5.0 [2.0-7.25]	0.722
(N=77)	Variant types	SNV	5.0 [4.0-6.0]	5.0 [3.0-6.25]	4.5 [2.0-7.0]	0.614
		INS	0.0 [0.0-0.0]	0.0 [0.0-0.0]	0.0 [0.0-0.0]	0.356
		DEL	0.0 [0.0-1.0]	0.5 [0.0-1.0]	0.5 [0.0-1.0]	0.194
	Impact of variants	High	0.0 [0.0-0.0]	0.0 [0.0-0.0]	0.0 [0.0-2.5]	0.381
		Moderate	4.0 [3.0-5.0]	4.5 [3.0-7.0]	5.0 [2.0-6.25]	0.299
		Low	1.0 [1.0-1.0]	0.0 [0.0-1.0]	0.0 [0.0-1.0]	<0.001
		Modifier	0.0 [0.0-0.0]	0.0 [0.0-0.0]	0.0 [0.0-0.0]	0.373
	SNV class	Transition	2.0 [1.75-3.0]	3.0 [ 1.75-3.00]	2.0 [ 1.00-3.00]	0.838
		Transversion	3.0 [2.0-3.0]	2.5 [2.0-4.0]	3.0 [ 1.00-4.00]	0.865
		Ti/Tv ratio	0.80 [0.670-1.330]	0.830 [0.500-1.330]	0.9250 [0.3715-1.0000]	0.730

*P-value was estimated by Kruskal-Wallis Test.

**Table 3:** Univariate and multivariate analyses of DFS in the study cohort.

Variables		Univariate analyses			Multivariate analyses		
		HR	95% CI	P-value	HR	95% CI	P-value
Age	<65	1	reference	0.778	1	reference	0.089
	≥65	0.884	0.375-2.086		2.806	0.854-9.220	
Sex	Male	1	reference	0.450	1	reference	0.298
	Female	1.428	0.566-3.602		0.42	0.082-2.151	
Smoking status	Never smoker	1	reference	0,422	1	reference	0.292
	Smoker	1.556	0.529-4.575		0.354	0.051-2.447	
Number of subclones	1	1	reference	<0.007	1	reference	0.012
	≥2	3.393	1.391-8.283		5.448	1.451-20.461	
pStage	I	1	reference		1	reference	
	II	12.675	1.603-100.220	<0.016	10.737	1.270-90.757	0.029
	III	16.048	2.061-124.94	<0.008	5.448	1.451-20.461	0.012

**Table 4:** Comparison of variants according to stage in the TCGA-LUAD cohort with mEGFR.

			Stage I (n=20)	Stage II (n=12)	Stage III (n=11)	Stage IV (n=2)	P-value**
TCGA-mEGFR	Total variant		41.5 [31.75-83.5]	60.5 [36.3-134.3]	53 [45.0-63.5]	40.5 [30.8-50.3]	0.663
(N=45*)	Variant types	SNV	38.0 [29.5-79.5]	58.0 [35.5-129.5]	52.0 [41.0-60.5]	39.0 [29.5-48.5]	0.627
		INS	0.0 [0.0-1.0]	0.0 [0.0-1.0]	0.0 [0.0-0.5]	0.0 [0.0-0.0]	0.564
		DEL	3.0 [2.0-3.3]	2.0 [1.0-3.5]	2.0 [1.0-3.0]	1.5 [1.3-1.8]	0.352
	Impact of variants	High	3.0 [3.0-8.3]	6.0 [2.8-10.3]	5.0 [2.5-6.5]	4.5 [3.8-5.3]	0.945
		Moderate	25.5 [18.8-45.0]	36.5 [23.8-86.5]	35.0 [23.5-38.5]	23.5 [17.3-29.8]	0.571
		Low	9.5 [6.0-18.0]	13.0 [8.3-25.3]	11.0 [8.5-17.0]	9.5 [7.8-11.3]	0.803
		Modifier	5.0 [3.8-8.3]	4.5 [3.0-11.0]	4.0 [2.5-9.0]	3.0 [2.0-4.0]	0.741
	SNV class	Transition	22.0 [17.5-37.3]	33.0 [20.8-50.3]	28.0 [ 20.5-38.0]	21.5 [14.8-28.3]	0.617
		Transversion	17.0 [10.0-33.5]	21.5 [15.8-74.8]	20.0 [ 16.5-29.0]	17.5 [ 14.8-20.3]	0.812
		Ti/Tv ratio	1.08 [0.73-1.78]	1.17 [0.81-1.60]	1.33 [0.82-2.07]	1.10 [0.88-1.31]	0.920

*Among 48 TCGA cases with EGFR-TKI sensitizing mutation, TCGA-17-Z032 and TCGA-17-Z047 was removed because stage is not available, and TCGA- 55-8506 was removed because of excessive number of mutations. **P-value was estimated by Kruskal-Wallis Test.

## Abbreviations

CNV: copy-number variation
DFS: disease-free survival
EGFR: epidermal growth factor receptor
mEGFR: EGFR-TKI sensitizing mutation
NGS: next-generation sequencing
OS: overall survival
TKI: tyrosine kinase inhibitor
VEP: variant-effect predictor
WES: whole-exome sequencing
