# Supplementary material for: Tumor clonal status predicts clinical outcomes of lung adenocarcinoma with EGFR-TKI sensitizing mutation
Source: J Cancer. 2019 Aug 29;10(22):5549–56. doi: 10.7150/jca.32897 (PMC6775702; doi:10.7150/jca.32897)
Supplement: Supplementary file 1 — Supplementary figures and tables. [file jcav10p5549s1.pdf]

Supplementary figure 1

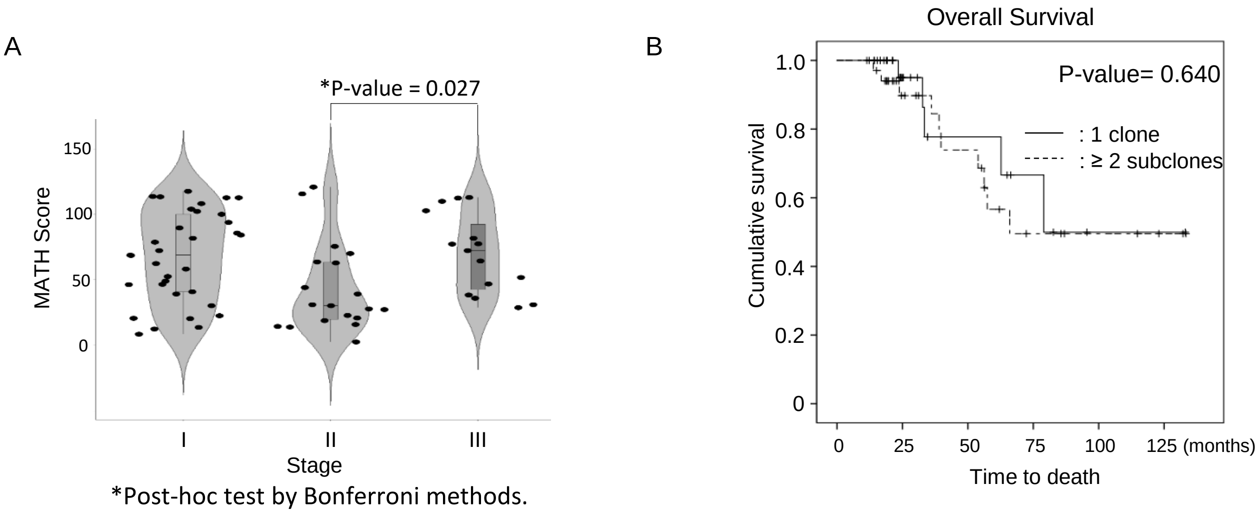

Supplementary figure 1. Comparison of (A) MATH score according to the stage, and (B) OS according the number of subclones in the study cohort. (A) When the MATH scores were compared according the stages, there was statistically significant difference of the MATH score according among the Stage (P-value 0.025. Kruskal-Wallis rank-sum test). The cases with Stage II showed lower MATH score than those with III (P-value = 0.027, by post-hoc test by Bonferroni methods). (B) The study cohort was classified into two groups, where the primary tumors consisted of either one clone and two or more subclones, and OS were compared using the Kaplan-Meier estimator. There was no difference between the two groups. P-values were obtained by the log-rank test.

Supplementary figure 2

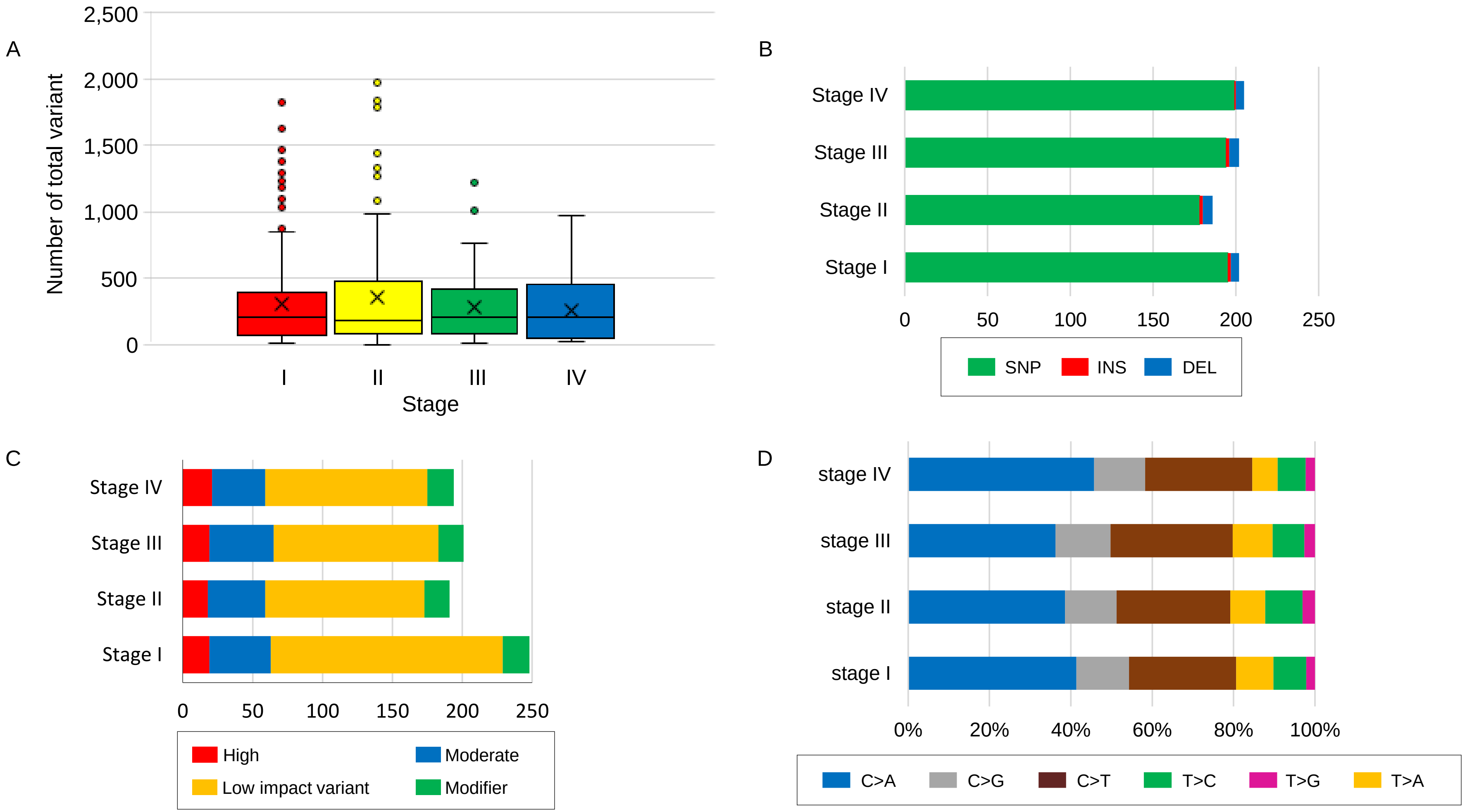

Supplementary figure 2. Comparison of mutational characteristics according to the stage in the entire TCGA-LUAD cases. Using the 502 TCGA-LUAD cases, which had both mutation information and clinical information, the differences in (A) the number of total mutations, and (B) those categorized into SNV, INS, and DEL were compared according to stage. (C) All mutations detected were classified according to VEP—high, moderate, low impact variant, and modifier—and compared according to the stage. (D) A histogram showing substitution type of SNVs per individual case. (A~D) The number of total mutations and that of subcategorized mutations were not statistically significant according to stage. The difference was estimated by the Kruskal-Wallis rank-sum test.

Supplementary figure 3

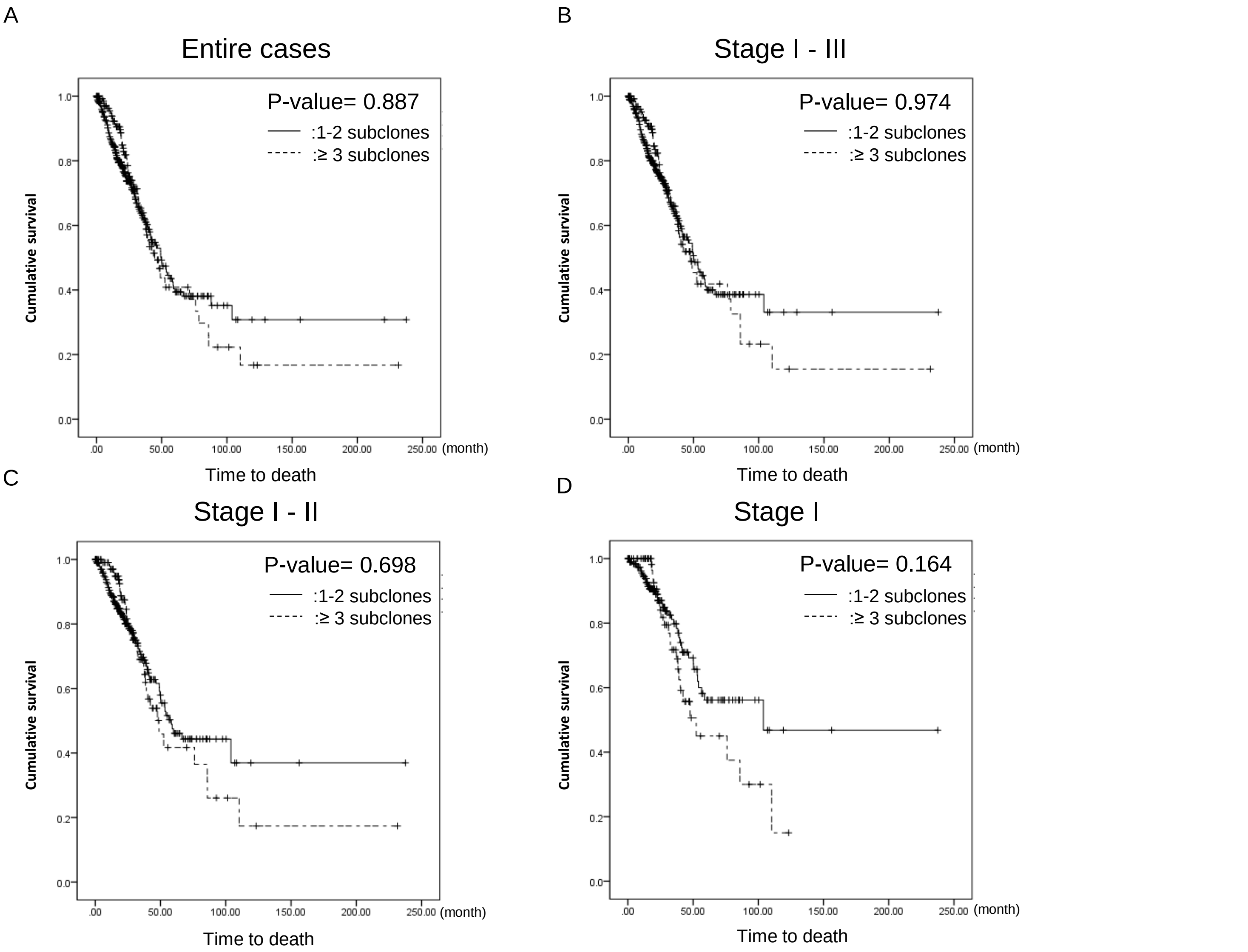

Supplementary figure 3. Survival analysis according to the number of subclones in TCGA-LUAD cases. 502 TCGA-LUAD cases into two groups, having one or two subclones and having three or more subclones, and OS was compared between the two groups. (A) entire TCGA-LUAD cases at stages I–IV, (B) those at stages I–III, (C) those at stages I –II, and (D) those at stage I. P-values were obtained by the log-rank test.
